# Differential Expression of Dickkopf 1 and Periostin in Mouse Strains with High and Low Bone Mass

**DOI:** 10.3390/biology11121840

**Published:** 2022-12-16

**Authors:** Katharina Kerschan-Schindl, Victoria Schramek, Maria Butylina, Ursula Föger-Samwald, Peter Pietschmann

**Affiliations:** 1 Department of Physical Medicine, Rehabilitation and Occupational Medicine, Medical University of Vienna, 1090 Vienna, Austria; 2 Institute of Pathophysiology and Allergy Research, Center for Pathophysiology, Infectiology and Immunology, Medical University of Vienna, 1090 Vienna, Austria

**Keywords:** C57BL/6J mice, C3H/J mice, sclerostin, dickkopf 1, periostin

## Abstract

**Simple Summary:**

Cells embedded in bone, so-called osteocytes, regulate bone metabolism by releasing various factors. Mouse studies are used both to investigate the background of various diseases and during the development of drugs. This is particularly relevant for osteoporosis, a disease characterized by a reduction of bone mass and an increased risk of bone fracture. The aim of this project was to investigate the differences in the expression of factors relevant for bone metabolism by osteocytes in two different mouse strains (C57BL/6J mice with low bone mass and C3H/J mice with high bone mass). The osteocytes’ expression of the investigated factors revealed strain- and age-associated differences. This study shows that the expression of specific factors by osteocytes reflects strain-related differences of bone properties.

**Abstract:**

By expressing different genes and proteins that regulate osteoclast as well as osteoblast formation, osteocytes orchestrate bone metabolism. The aim of this project was the evaluation of the differences in the osteocytes’ secretory activity in the low bone mass mouse strain C57BL/6J and the high bone mass strain C3H/J. The femura of eight- and sixteen-week-old male C57BL/6J and C3H/J mice—six animals per group—were analyzed. Using immunohistochemistry, osteocytes expressing dickkopf 1, sclerostin, periostin, fibroblast growth factor 23 (FGF23), and osteoprotegerin were detected. By means of the OsteoMeasure-System, 92.173 osteocytes were counted. At the age of eight weeks, approximately twice as many cortical and trabecular osteocytes from the C57BL/6J mice compared to the C3H/J mice expressed dickkopf 1 (*p* < 0.005). The number of cortical osteocytes expressing sclerostin was also higher in the C57BL/6J mice (*p* < 0.05). In contrast, the cortical and trabecular osteocytes expressing periostin were twice as high in the C3H/J mice (*p* < 0.005). The dickkopf 1 expressing osteocytes of the C57BL/6J mice decreased with age and showed a strain-specific difference only in cortical bone by 16 weeks of age (*p* < 0.05). In the C3H/J mice, the amount of osteocytes expressing periostin tended to increase with age. Thus, strain-related differences were maintained in 16-week-old rodents (*p* < 0.005). No strain-specific differences in the expression of FGF23 or osteoprotegerin in the cortical compartment could be detected. This experimental study showed that the osteocytes’ protein expression reflects differences in bone characteristics and strain-related differences during skeletal maturation. Besides the osteocytes’ expression of sclerostin, their expression of dickkopf 1 and periostin seems to be important for bone properties as well.

## 1. Introduction

Although osteocytes comprise more than 90% of all bone cells, their role in bone metabolism has been underestimated for a long time. Osteocytes, which are embedded within the mineralized bone matrix, have several different functions: they regulate osteoclasts as well as osteoblasts, regulate phosphate and calcium homeostasis, are the main mechanosensors, and target organs such as the kidney [1].

Like the conductor of an orchestra, these endocrine cells express different genes and proteins and, thereby, play an important role in bone metabolism. For instance, in the case of unloading, osteocytes increase their secretion of sclerostin, an inhibitor of osteoblast differentiation [2] that, meanwhile, is the target of a bone-specific medication approved for osteoporosis. Dickkopf 1 (DKK1), the second inhibitor of the Wnt/ß-catenin signaling pathway necessary for osteoblast differentiation, is secreted by osteocytes as well [3]. Besides these antagonists of osteoblast differentiation, osteocytes also express periostin (PSTN), a protein essential for bone formation [4]. The osteocyte is the main source of fibroblast growth factor 23 (FGF23), an important regulator of phosphate and vitamin D metabolism [5]. The receptor activator kappa B (RANK)/RANK ligand (RANKL)/osteoprotegerin (OPG) system regulating osteoclast formation is also dependent on the osteocytes’ functions, as these multifunctional endocrine cells express RANKL and the decoy receptor OPG [6].

Animal models, especially inbred mouse strains, provide important insights into bone health and disease. Using micro-computed tomography, we previously evaluated age- and strain-related differences in the microstructure of different mouse strains: C3H/J mice had more favorable cortical bone parameters, whereas C57BL/6J mice had more favorable trabecular bone parameters [7]. All together, C57BL/6J mice can be used as a low bone mass strain for osteoporosis research, whereas C3H/J mice are supposed to be a high bone mass strain. Age-related changes in bone structure equivalent to humans and degeneration of osteocytes have been shown in C57BL/6J mice [8].

The aim of this project was to evaluate osteocyte-derived molecules, which are related to bone formation and, thus, may play an important role for the molecular basis responsible for strain-related differences in bone characteristics of C57BL/6J and C3H/J mice. Knowledge of the osteocytes’ expression of proteins involved in bone metabolism may be the basis for future studies evaluating further therapeutic strategies. We tested the hypothesis that the expression of inhibitors of the Wnt/ß-catenin signaling pathway would be higher in the C57BL/6J mice—the low bone mass strain—compared to the C3H/J mice. On the other hand, PSTN expression was supposed to be higher in the C3H/J mice. Additionally, we counted the number of osteocytes positive for FGF23 and OPG.

## 2. Mice, Materials, and Methods

### 2.1. Animals

Male C57BL/6J (Abteilung für Labortierkunde und Genetik; Himberg, Austria) and male C3H/J (Charles River, Sulzfeld, Germany) mice were housed in groups of two to six mice per cage at the animal facility of the Department of Pathophysiology and Allergy Research, Medical University of Vienna. The light–dark cycle was 12:12 h. The animals had free access to a standard rodent diet (LASQCdiet^®^ Rod 16, Auto; LASvendi GmbH, Soest, Germany) and water. At the age of eight and sixteen weeks, six animals per mouse group were euthanized by carbon dioxide asphyxiation. All procedures were performed in accordance with the national and institutional laws and regulations.

### 2.2. Sample Preparation

The femura were dissected free of musculature and connective tissue. The bones were preserved in formaldehyde for 24 h at 4 °C. The bones were decalcified by incubation in trisaminomethane/ethylene-diamine-tetra acid (TRIS/EDTA) at 4 °C for three days. Subsequently, the bones were dehydrated for one hour in 80% ethanol and for another hour in 96% ethanol. The final dehydration step was an incubation in xylol for 30 min. Afterwards, the bones were embedded in different wax baths (paraffin with a melting point between 52 and 54 °C as well as paraffin with a melting point between 56 and 58 °C) at 60 °C for a total of 150 min. The paraffin-infiltrated femura were put in molds and filled up with liquid paraffin. After solidification of the paraffin, 10 slices of 5 to 8 µm, depending on sample porosity, were cut with a Microm HM 355S (Thermo Scientific^TM^) and stained using immunohistochemical methodology (IHC).

### 2.3. Immunohistochemistry

The slides were incubated with an anti-DKK1 antibody (ab61034, Abcam, Cambridge, UK), anti-SOST antibody (ab85799, Abcam, Cambridge, UK), anti-PSTN antibody (ab92460, Abcam, Cambridge, UK), anti-FGF23 antibody (LS-C293901, LSBio, Seattle, WA, USA), anti-OPG antibody (ABIN668556, Antibodies-online, Aachen, Germany), and an isotype control antibody (ab27478, Abcam, Cambridge, UK) overnight at 4 °C. The dilution used in each case was 1:100 with blocking buffer. In the second step, 100 µL of the enzyme-activated secondary antibody (anti-rabbit polymer) bound to the primary antibody and was visualized with the addition of hydrogen peroxide (H_2_O_2_) and the chromogen DAB (3.3′-diaminobenzidin). The time of incubation was one to four minutes. After the secondary step, the slides were counterstained with hematoxylin for the visualization of the cell nuclei.

### 2.4. Histomorphometry

**Figure 1:**
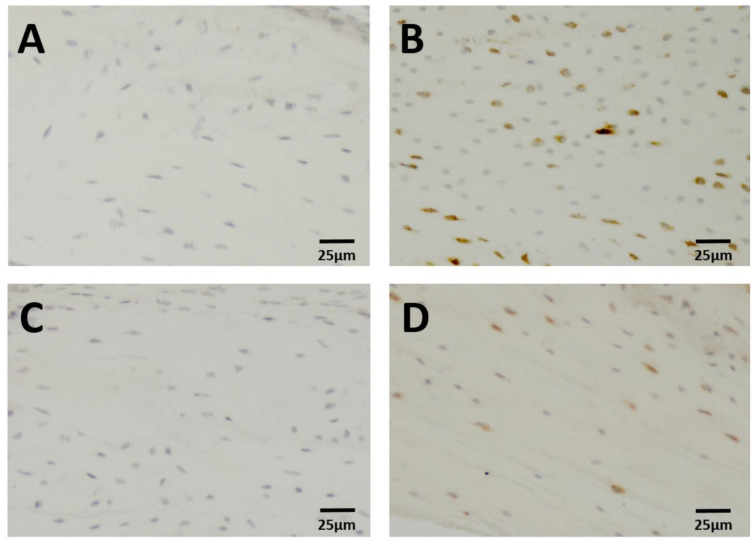
Immunohistochemistry: DKK1 expression in cortical bone of 8-week-old mice (**A**) C57BL/6J: negative control, (**B**) C57BL/6J: osteocytes positive for DKK1 C57BL/6J, (**C**) C3H/J: negative control, (**D**) C3H/J: osteocytes positive for DKK1. Brown-stained osteocytes: positive signal. Osteocytes stained with hematoxylin only: negative signal.

Computer-assisted image analysis was performed with the OsteoMeasure-System (OsteoMetrics, Atlanta, GA, USA). Using a 20× magnification, we counted osteocytes that were positive for the proteins DKK1 and PSTN, both trabecular and cortical, in each of 20 fields with a size of 0.15 mm^2^. In addition, osteocytes positive for SOST, FGF-23, and OPG were counted in five to ten fields of cortical bone. Osteocytes with a detectable staining signal were defined as positive for the respective protein; we did not assess the differences in staining intensity (Figure 1). The measuring area of the trabecular compartment was set at the distal femur with a distance of approximately 250 µm from the growth plate. The region of interest for cortical analyses was located at the diaphysis and included the third quarter of the femur.

In addition to the quantification of immunohistochemistry, static trabecular histomorphometry following the ASBMR guidelines [9] was performed. BV/TV (bone volume/tissue volume), Tb.Th (trabecular thickness), TbSp (trabecular separation), and TbNb (trabecular number) were evaluated.

### 2.5. Statistical Analysis

Descriptive statistics were performed with the GraphPad Prism-Software version 5.0 (Graphpad Software, Inc., San Diego, CA, USA). The two-way analysis of variance (2-way ANOVA) was used to evaluate the influence of strain and age on the osteocytes’ protein expression. The Bonferroni test was used as a post hoc test. *p*-values ≤ 0.05 were considered statistically significant.

## 3. Results

All together, 92.173 osteocytes were counted. In the eight-week-old animals, the percentage of cortical as well as trabecular osteocytes expressing DKK1 was distinctly higher in the C57BL/6J mice than in the C3H/J mice (*p* < 0.0001 and *p* = 0.0004, respectively). The amount of cortical osteocytes expressing SOST was also significantly higher in the C57BL/6J mice than in the C3H/J mice at eight weeks of age (*p* = 0.0032). By the age of 16 weeks, the differences in the expression of the inhibitors of the Wnt/ß-catenin signaling pathway diminished and were only significant for the osteocytes positive for DKK1 in the cortical area (*p* = 0.0458). Concerning PSTN, the expression of cortical and trabecular osteocytes was more than twice as high in the C3H/J mice as in the C57BL/6J mice at the age of eight weeks (*p* < 0.0001 and *p* = 0.0043, respectively) and sixteen weeks (*p* < 0.0001 and *p* = 0.0022, respectively). No strain-specific differences in the expression of FGF23 or OPG in the cortical compartment could be detected (Table 1).

**Figure 2:**
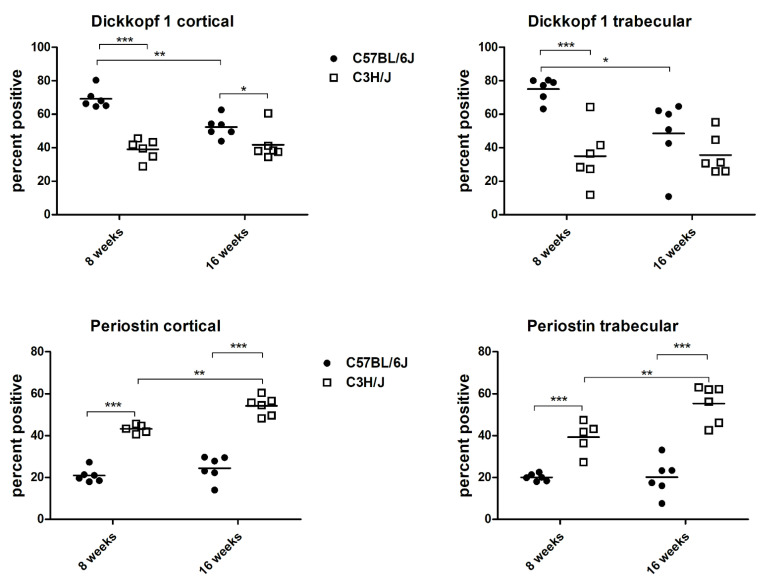
Cortical and trabecular osteocytes positive for dickkopf 1 and periostin; * *p* < 0.05, ** *p* < 0.01, *** *p* < 0.001.

Besides the strain-specific difference, the two-way analysis of variance detected a significant decrease in cortical and trabecular DKK1 in the C57BL/6J mice from eight weeks to sixteen weeks of age (*p* < 0.01 and *p* < 0.05, respectively; Figure 2).

**Figure 3:**
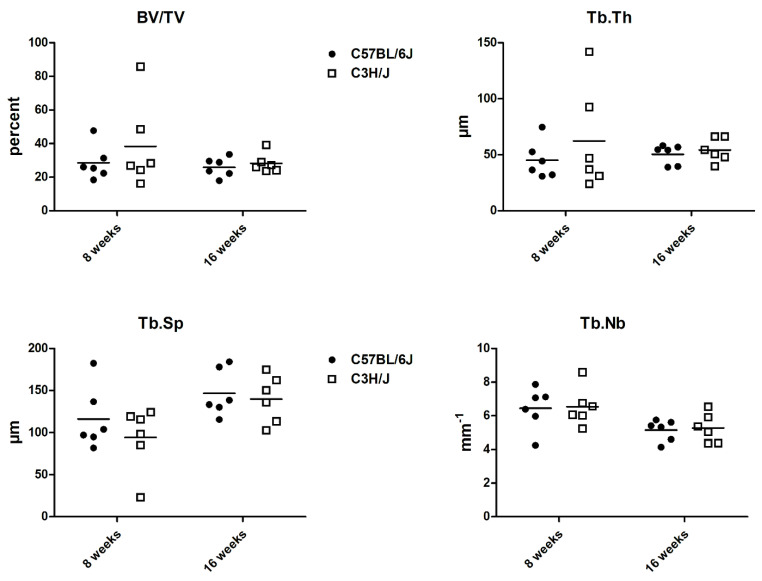
Histomorphometric data of C57BL/6J and C3H/J mice at 8 and 16 weeks of age. BV/TV: bone volume/tissue volume; Tb.Th: trabecular thickness; TbSp: trabecular separation; TbNb: trabecular number.

Although histomorphometry did not reveal significant differences between the two mouse strains, at eight weeks of age, BV/TV and Tb.Th tended to be higher in the C3H/J mice. (Figure 3).

## 4. Discussion

This study detected strain-specific differences in the osteocytes’ DKK1, SOST, and PSTN expression. The importance of DKK1 concerning bone formation has already been highlighted in some studies. Using a transgenic animal model, Codlitz and coauthors [10] found that, despite the fact that the majority of DKK1 originates from osteoprogenitors, the amount of DKK1 released from mature osteogenic cells is sufficient to modulate bone mass. An experimental study evaluating DKK1 knockout mice proved that the increased bone formation associated with DKK1 deficiency cannot be compensated by upregulation of sclerostin [11]. According to our hypothesis, the number of osteocytes positive for the Wnt-inhibitor DKK1 was significantly higher in the C57BL/6J mice, which was the strain with the less-favorable bone properties. The importance of DKK1 for bone homeostasis is also highlighted by the findings that loss of DKK1 partially reduces altered bone volume associated with hypo- as well as hyperthyroidism [12] and significantly reduces alveolar bone loss in mice with experimentally induced periodonditis [13]. Most human studies evaluated potential associations between serum levels of DKK1 and BMD. According to a narrative review [14], there is no consistent evidence of an association between serum levels of DKK1 and BMD. In postmenopausal women with a previous fragility fracture [15] and in elderly women with a recent femoral neck fracture [16], a positive correlation between protein DKK1 levels in the bone and BMD was detected. These opposite results can probably be explained by the different methodology used. They analyzed DKK1 levels in bone powder, whereas we counted the number of osteocytes positive for DKK1. This study evaluated mice during their adolescence, a period of life characterized by rapid bone growth and development. So far, no studies evaluating the association of DKK1 levels in bone and BMD during human adolescence exist. The strain-specific difference of this study’s mice diminished with age, showing significance only for the expression of DKK1 in the cortical compartment by 16 weeks. We have previously shown that the cortical and trabecular bone development of C57BL/6J mice peaked at 24 weeks, whereas C3H/J mice reached maximal skeletal morphological and biomechanical properties before 16 weeks of age [7]. From a physiologic point of view, a higher expression of DKK1, the antagonist of the Wnt ß-catenin pathway, may go hand-in-hand with a lower amount of bone modeling after skeletal maturity. This could be responsible for the diminished strain-specific difference in the expression of DKK1 with increasing age. The fact that the C3H/J mice, as a high bone mass strain, had significantly lower DKK1 expressions than the C57BL/6J mice in cortical and trabecular bone fits well with the data by MacDonald and coauthors who investigated DKK1-mutant mice that expressed low amounts of DKK1 and wild-type mice. They showed that DKK1 expression correlated negatively with cortical and trabecular bone volume [17]. The difference in osteocytic DKK1 expression between the different mouse strains may indeed be relevant for preclinical testing of DKK1 inhibitors, which are intensively studied, especially for cancer treatment [18].

The importance of SOST in the regulation of bone turnover has been demonstrated in patients with high bone mass that lack SOST expression and verified in SOST knockout mice; their bone formation was increased [19]. Because of the positive data of the Fracture Study in Postmenopausal Women with Osteoporosis (FRAME) [20,21], the SOST antibody treatment nowadays is used in clinical routine. The authors of a previous investigation detecting low serum sclerostin levels in subjects with low BMD assumed that the low sclerostin expression was due to reduced bone volume [22]. In young subjects with a mean age of 23 years, circulating levels of sclerostin did not correlate with BMD [23]. This experimental study evaluating mice during bone growth showed that the expression of SOST evaluated in the cortical compartment was significantly lower in the high bone mass strain at eight weeks of age. These results concur with the physiological function of SOST and the increased bone formation of 10-week-old SOST-deficient mice [24].

The osteocytes’ expression of PSTN was significantly higher in the C3H/J mice compared to the C57BL/6J mice. This finding stresses the strain-specific differences of bone mass. Previous experimental studies showed that the lack of PSTN has a detrimental effect on cortical and trabecular BMD as well as microarchitecture [25,26,27]. PSTN has also been shown to be an indispensable protein for the anabolic response following mechanical stimuli [25] as well as intermittent parathyroid hormone therapy [28] and to promote the estrogen-induced osteogenic differentiation of bone marrow stromal cells via activation of the Wnt/ß-catenin signaling pathway [29]. Since PSTN plays a complex role in bone anabolism, the stronger PSTN expression in the high bone mass strain of eight- and sixteen-week-old rodents seems appropriate. The fact that the number of osteocytes positive for PSTN showed strain-specific differences in the cortical as well as the trabecular compartment fits two previous studies evaluating PSTN-/-mice, which showed deficits in cortical and trabecular bone compared to the wild-type littermates [25,26]. The high amount of osteocytes expressing PSTN in the C3H/J mice may also be the driving force for the high SOST expression in the C3H/J mice; an experimental investigation has shown that PSTN is required for SOST inhibition when inducing an anabolic response [25].

The lack of a strain-related difference of FGF23 expression in the cortical compartment is in line with the previous literature. Almost all studies so far have found no correlation between FGF23 and BMD in humans [14]. A recent review concluded that only supraphysiological levels of FGF23 cause abnormal bone formation [30].

As a decoy receptor of RANKL, OPG plays an important role in bone metabolism. Whyte et al. described a defect in the gene encoding OPG and undetectable serum levels of OPG in two patients with juvenile Paget’s disease, which is a disease associated with low BMD and a high risk of fracturing [31]. A recent cross-sectional study evaluating 156 patients with rheumatoid arthritis, however, did not find a difference in serum OPG levels between those with and those without osteoporosis [32]. Our evaluation of high and low bone mass mice also could not detect a difference in the cortical osteocytes’ OPG expression.

In line with previous microCT (micro-computed tomography) data [7], histomorphometric evaluation of the bone microstructure seemed to be more favorable in the C3H/J mice. However, no significant strain-specific differences could be evaluated in the two-dimensional method used in this study.

We describe the differences of factors relevant for bone formation in the two mouse strains. These fit mechanistically. Since we did not perform an intervention study, we can only describe a relation but cannot prove a causal relationship. Further limitations of the present study are worthy to mention. The osteocytes’ SOST expression was only evaluated in the cortical compartment. Since there is a huge amount of data on SOST, we did not further assess SOST expression. According to an experimental study, estrogen deficiency induced by ovariectomy led to an increase in SOST expression in cortical bone [33]. In postmenopausal osteoporotic women, an evaluation of the trabecular and cortical bone of trans-iliac crest bone biopsies revealed that only cortical bone matrix levels of SOST correlated with bone mass and strength [15]. Since no strain-specific differences in the expression of FGF23 and OPG could be detected in the cortical compartment, we decided to omit the estimation of the trabecular compartment in this regard. Another limitation is that we investigated only two age groups of mice. We decided to evaluate eight-and sixteen-week-old animals due to the good discriminability between strains at these ages. Because of the exploratory character of this study, we chose six mice of each strain and each age.

## 5. Conclusions

Our findings of strain- and age-related differences in the osteocytes’ protein expression seem to reflect variations in bone characteristics during skeletal maturation. Despite the availability of a huge amount of studies evaluating SOST, we should not forget the importance of DKK1 and PSTN expression by osteocytes, which are important for bone properties as well.

## Figures and Tables

**Table 1:** Immunohistochemistry: Osteocytes positive for different proteins as absolute values of the total number of counted osteocytes and as percentages.

Variable	C57BL/6J—8 we	C3H/J—8 we	C57BL/6J—16 we	C3H/J—16 we
DKK1 cortical	4121/6024	3893/9888	2124/4031	1624/4004
DKK1 cortical %	69 ± 6	39 ± 6 ***	52 ± 6	42 ± 9 *
DKK 1 trabecular	2357/3173	1312/3838	775/1614	738/2147
DKK 1 trabecular %	75 ± 7	35 ± 18 **	49 ± 20	36 ± 12
SOST cortical	1257/2620	702/2347	1170/2572	862/2337
SOST cortical %	49 ± 9	30 ± 8 *	45 ± 8	39 ± 12
PSTN cortical	789/3702	1789/4160	858/3455	1939/3622
PSTN cortical %	21 ± 3	43 ± 2 ***	24 ± 6	54 ± 5 ***
PSTN trabecular	327/1653	645/1779	327/1688	926/1691
PSTN trabecular %	20 ± 2	39 ± 8 ***	20 ± 9	55 ± 9 **
FGF23 cortical	1182/2307	1289/2387	1010/2462	1344/2430
FGF23 cortical %	54 ± 16	54 ± 5	42 ± 13	55 ± 8
OPG cortical	1938/3443	2758/5265	1888/3598	1681/3051
OPG cortical %	58.88 ± 8.77	54.66 ± 14.04	53.51 ± 7.88	55.73 ± 10.94

DKK1: dickkopf 1; SOST: sclerostin; PSTN: periostin; FGF23: fibroblast growth factor 23; OPG: osteoprotegerin; mean ± SD, Differences between strains at 8 and 16 weeks, respectively, as * *p* < 0.05, ** *p* < 0.005, *** *p* < 0.001.

## Data Availability

The data of this study are available from the corresponding author upon reasonable request.
